# Prevalence and Perceptions of Dietary Supplement Use Among Nepalese Gym-Users: An Observational Study

**DOI:** 10.31729/jnma.8957

**Published:** 2025-04-30

**Authors:** Yoveen Kumar Yadav, Niraj Sapkota, Rahul Jha, Amit Chand, Kunal Pathak, Garima Thakur, Sunil Kunwar

**Affiliations:** 1 Hope International College, Mahalaxmisthan, Lalitpur, Nepal; 2 Patan Academy of Health Sciences, Lagankhel, Lalitpur, Nepal; 3 Nobel Medical College, Kanchanbari, Biratnagar, Nepal

**Keywords:** *dietary supplements*, *health knowledge, attitudes, practice*, *muscle strength*, *Nepal*, *resistance training*

## Abstract

**Introduction:**

Dietary supplements are widely used by gym-goers to enhance physical appearance and performance, but their inappropriate use poses potential health risks. This study aimed to assess the patterns, motivations, and knowledge of dietary supplement use among gym users in the Lamjung District of Nepal.

**Methods:**

This was an observational cross-section study conducted from December 16, 2020, to January 13, 2021, among 214 gym users. Data were collected using a structured questionnaire based on the Health Belief Model (HBM) framework. Descriptive statistics were used to analyze the data.

**Results:**

Among 214 gym users surveyed, 70 (32.71%) reported consuming dietary supplements. Protein and amino acids were used by 49 (70%) of the supplement consumers. The primary motivation for supplement use was muscle mass enhancement 62 (88.57%), followed by increasing strength and power 46 (65.71%). Peers influenced 48 (68.57%) of users, while advertisements had minimal influence 3 (4.29%). Knowledge about supplements showed, 141 (65.89%) believing supplements could contribute to cancer and 126 (58.88%) agreeing that Nepal lacks effective quality control for these products.

**Conclusions:**

Many gym users in Nepal, mostly male students aged 23-28, take dietary supplements — mainly proteins and amino acids — to boost muscle mass, often influenced by peers. The findings reveal gym users perceive high benefits but low health risks from supplements, with peer influence outweighing expert guidance.

## INTRODUCTION

Dietary supplements are known as an independent food category consisting of more than one dietary component, including vitamins, minerals, protein, calcium, herbs, and meal substitutes.^1^ They are available in the form of tablets, capsules, powders, or pills. The use of dietary supplements has increased significantly to decrease susceptibility to diseases, improve personal health, and avert nutritional deficiencies.^2,3^ However, inappropriate use poses potential health risks, including obesity, cancer, and cardiovascular diseases.^3^

Worldwide, studies have shown a high prevalence of hormone and dietary supplement use by gym users who are in search of the perfect body and enhanced physical appearance.^4,5^ However, research highlights the long-term harmful effects of dietary supplements, and withdrawal from their use has been linked to severe consequences. Studies in different countries have even reported deaths associated with supplement use.^4,5^

Research on dietary habits and supplement use highlights diverse behaviours and significant gaps in knowledge. To our knowledge, there is no existing data on dietary supplement use among gym users in Nepal. This study focuses specifically on gym users in the Lamjung District of Nepal to assess their supplement use patterns and related factors.

## METHODS

This was an observational cross-section study from 16 December 2020 to 13 January 2021. The study site was gym centres of Lamjung, Nepal. Ethical approval was taken from Institutional Review Committee (IRC) of Nobel College (Reference number: MPHIRC328/2020). Informed consent was obtained from study participants and gym owners. There are two gyms operating in Lamjung District.

A complete enumeration of all sampling units was performed. This approach ensured that all eligible participants were included, providing comprehensive data on supplement use trends. There were 214 available respondents for the interview. Gym user people who are in normal state of health and who are regularly engaged in the gym for at least one month were included in the study while the users of age less than 18 years old, with any known disease condition and unwilling to participate in the study were excluded from the study.

This study utilized a structured questionnaire to assess perceptions and attitudes toward dietary supplements, incorporating two different tools: a 3-point Likert scale-based questionnaire and an HBM-based questionnaire. These tools were designed to capture key factors influencing supplement use, including perceived susceptibility, severity, benefits, barriers, and cues to action.

The questionnaire was divided into four sections: demographic information (age, gender, education, occupation, and income level), exercise and supplement use patterns (frequency and duration of gym sessions, types of supplements used, dosage, and reasons for usage), knowledge and awareness assessment (understanding of supplement benefits, risks, and regulatory guidelines), and constructs of the HBM (perceived susceptibility, severity, benefits, barriers, and cues to action). The content was developed based on an extensive literature review, and the questionnaire was validated by faculty members of Nobel College to ensure content accuracy.

To ensure content validity, the questionnaire was developed with input from experts in public health, nutrition, and behavioural sciences. Face validity was assessed through a pilot study conducted with a representative sample of the target population. Participants provided feedback on the clarity, relevance, and comprehensibility of the questions, and minor refinements were made before final implementation.

Construct validity was evaluated through exploratory factor analysis (EFA), confirming that the items aligned with the theoretical framework of the HBM. Internal consistency reliability was measured using Cronbach's alpha, with a threshold of >0.7 considered acceptable.

Pre-testing was conducted with 10% of the total sample size to familiarize researchers with the tools, ensure proper sequencing of questions, and build researcher confidence. To reduce response bias, the questionnaire included both positively and negatively worded items where applicable. Anonymous participation was ensured, and participants were assured of confidentiality to minimize social desirability bias.

The final version of the questionnaire was reviewed and approved by the institutional ethics committee, and informed consent was obtained from all participants before data collection.

Written informed consent was taken from all participants before data collection, ensuring confidentiality and voluntary participation. Data were entered using EpiData and analyzed using the Statistical Package for Social Sciences (SPSS). Descriptive statistics such as frequency and percentage distributions were used to summarize demographic characteristics

## RESULTS

Out of the total 214 participants, 94 (43.93%) were in the 23-28 age group, 53 (24.8%) in the 17-22 age group, and 2 (0.93%) were aged 41 and older. The study population included 199 (92.99%) males. Regarding marital status, 114 (53.27%) were unmarried. For education level, 135 (64.95%) had secondary-level education, while 75 (35.05%) had completed graduation (Table 1).

**Table 1:** Distribution of the respondents by their age/sex composition (n=214).

Characteristic	Frequency (%)
**Age**	
17-22	53 (24.77%)
23-28	94 (43.93%)
29-34	54 (25.23%)
35-40	11 (5.14%)
41 and Older	2 (0.93%)
**Sex**	
Male	199 (92.99%)
Female	15 (7.01%)
**Education**	
Primary Level	-
Secondary Level	139 (64.95%)
Graduate and above	75 (35.05%)
**Marital Status**	
Married	100 (46.73%)
Unmarried	114 (53.27%)

**Figure 1:**
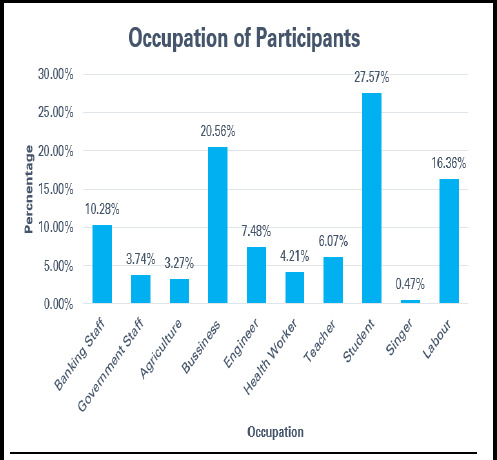
Occupation of the Participants (n=216).

Out of 214 participants, 59 (27.57%) were students, 44 (20.56%) were business professionals, and 35 (16.35%) were labourers. 9 (4.21%) of respondents worked in the health sector (Figure 1). The median monthly income was NPR 29,400, with 106 (49.5%) earning between NPR 20,000 and NPR 40,000. 62 (28.97%) gym users were unemployed and not engaged in income-generating activities.

Among 214 participants, 85 (39.72%) reported strength training as their exercise type, 52 (24.3%) engaged in balance exercises, 51 (23.83%) performed endurance exercises, and 26 (12.15%) practiced flexibility exercises.

Among the 214 respondents, 70 (32.71%) reported using dietary supplements, with 67 (95.71%) of these users being male and 3 (4.29%) female. Protein and amino acid supplements were used by 49 (70%) of supplement consumers, followed by multivitamins and minerals 13 (18.57%), isotonic drinks 6 (8.57%), and carbohydrate supplements 1 (1.43%). The primary reason for supplement use was muscle mass enhancement (62, 88.57%), while 46 (65.71%) cited increasing strength and power, 20 (28.57%) reported maintaining overall health, and 9 (12.86%) mentioned role modelling as their motivation. Regarding influences on supplement use, peers were cited by 48 (68.57%) respondents, gym instructors by 19 (27.14%), and advertisements by only 3 (4.29%).

Regarding knowledge of dietary supplement risks, 113 (52.80%) respondents disagreed that supplements increase health problem risks, while 33 (15.42%) agreed. When asked about supplement-related symptoms during irregular exercise, 105 (49.07%) disagreed they could cause headaches, compared to 58 (27.10%) who agreed (Table 2).

**Table 2:** Distribution of Respondents by Their Knowl­edge on Dietary Supplements (n=214).

Statement	Agree	Disagree	Don't Know
Dietary supplements lead to an increased risk of health problems.	33 (15.42)	113 (52.80)	68 (31.78)
Dietary supplements cause symptoms (e.g., headache) when exercise is done irregularly.	58 (27.10)	105 (49.07)	51 (23.83)
Dietary supplement intake is mostly used by men.	16 (7.48)	108 (50.47)	90 (42.06)
Dietary supplements can contribute to cancer.	141 (65.89)	55 (25.70)	18 (8.41)
After taking dietary supplements, gym users feel in-clined to do heavy exercise.	79 (36.92)	110 (51.40)	25 (11.68)
By age 18, the majority of gym users take dietary sup-plements.	18 (8.41)	133 (62.15)	73 (34.11)
High socioeconomic people take a large number of die-tary supplements.	5 (2.34)	110 (51.40)	99 (46.26)
Any type of physical activity is beneficial after dietary supplement intake.	109 (50.93)	95 (44.39)	10 (4.67)
Dietary supplements alone can prevent low muscle mass.	52 (24.30)	80 (37.38)	82 (38.32)
Dietary supplements are used to prevent nutritional deficiency.	89 (41.59)	87 (40.65)	38 (17.76)
Dietary supplements are used to increase energy.	12 (5.61)	77 (36.45)	125 (58.41)
There is no effective quality control on dietary supplements in Nepal.	126 (58.88)	1 (0.47)	87 (40.65)

Regarding perceived susceptibility, 200 (93.46%) participants (combined agree/strongly agree) believed gym users were likely to gain muscle mass from supplements. For perceived severity, 76 (35.51%) strongly believed supplement-related health problems could be serious. On benefits, 119 (55.60%) (combined agree/strongly agree) acknowledged supplements’ role in muscle building. For barriers, 89 (41.59%) strongly agreed cost was prohibitive. Regarding cues to action, 94 (43.92%) reported coach recommendations (combined agree/strongly agree) as influential, while only 13 (6.07%) strongly agreed parental support existed (Table 3).

**Table 3:** Distribution of Respondents by Their Knowledge on Dietary Supplements (n=214).

HBM Constructs	Statement	S.D	Disagree	Neutral	Agree	S.A
Perceived Susceptibility	The chances of getting more muscle mass are high due to dietary supplements.	12 (5.61)	56 (26.17)	4 (1.87)	60 (28.04)	82 (38.32)
	Because of body build, there is more likely to develop muscle mass.	1 (0.47)	-	61 (28.50)	91 (42.52)	61 (28.50)
	It is extremely likely that gym user will develop high muscle mass.	1 (0.47)	-	13 (6.07)	112 (52.34)	88 (41.12)
	There is a good chance that gym user will develop high muscle mass.	1 (0.47)	-	23 (10.75)	100 (46.73)	90 (42.06)
	Gym users are more likely than the average person to develop high muscle mass.	5 (2.34)	-	3 (1.40)	110 (51.40)	96 (44.86)
Perceived Severity	The thought of having side effects from dietary supplements scares gym user.	44 (20.56)	52 (24.30)	96 (44.86)	17 (7.94)	5 (2.34)
	Gym user's feelings about themselves would change if they got side effects from dietary supplements.	59 (27.57)	98 (45.79)	42 (19.63)	13 (6.07)	2 (0.93)
	The dietary supplements would cause carcinogenic effects in future.	56 (26.17)	64 (29.91)	66 (30.84)	20 (9.35)	8 (3.74)
	It would be very costly if gym user got health problems from dietary supplements.	47 (21.96)	88 (41.12)	62 (28.97)	14 (6.54)	3 (1.40)
	It would be very serious if gym user got health problems from dietary supplements.	76 (35.51)	58 (27.10)	76 (35.51)	3 (1.40)	1 (0.47)
Perceived Benefit	Regular dietary supplements prevent problems that would happen from exercise.	34 (15.89)	92 (42.99)	33 (15.42)	30 (14.02)	25 (11.68)
	Gym users feel better when they take dietary supplements.	20 (9.35)	88 (41.12)	39 (18.22)	50 (23.36)	17 (7.94)
	Regular intake of dietary supplements helps to build muscle mass.	35 (16.36)	57 (26.64)	3 (1.40)	80 (37.38)	39 (18.22)
	Regular dietary supplements also improve the way gym user's body looks.	52 (24.30)	41 (19.16)	23 (10.75)	57 (26.64)	41 (19.16)
	Gym users feel good about themselves when they take dietary supplements.	41 (19.16)	74 (34.58)	41 (19.16)	42 (19.63)	16 (7.48)
Perceived Barrier	Dietary supplements cost too much.	1 (0.47)	17 (7.94)	57 (26.64)	50 (23.36)	89 (41.59)
	Dietary supplements foods do not agree with gym users.	38 (17.76)	45 (21.03)	104 (48.60)	16 (7.48)	11 (5.14)
	Taking dietary supplements means changing gym user's diet which is hard to do.	18 (8.41)	71 (33.18)	84 (39.25)	30 (14.02)	11 (5.14)
	In order to eat more dietary supplements, gym users have to give up other foods that they like.	25 (11.68)	145 (67.76)	40 (18.69)	4 (1.87)	-
	Dietary supplements foods have too much cholesterol.	15 (7.01)	61 (28.50)	136 (63.55)	2 (0.93)	-
Cues to Action	Parents support me to buy dietary supplement.	112 (52.34)	87 (40.65)	9 (4.21)	4 (1.87)	2 (0.93)
	Friends support me to buy dietary supplement.	68 (31.78)	63 (29.44)	7 (3.27)	32 (14.95)	44 (20.56)
	Advertisements showed on TV promote to buy dietary supplement.	66 (30.84)	85 (39.72)	7 (3.27)	36 (16.82)	20 (9.35)
	Coach told me to use dietary supplement to increase body mass.	44 (20.56)	75 (35.05)	1 (0.47)	81 (37.85)	13 (6.07)
	Sport person guide me to use dietary supplement.	74 (34.58)	114 (53.27)	8 (3.74)	17 (7.94)	1 (0.47)

## DISCUSSION

This study provides an in-depth exploration of dietary supplement consumption among gym-goers in Nepal, focusing specifically on Lamjung District. The findings reveal that dietary supplements are widely used, particularly among young male adults engaged in strength training. The primary reason gym-goers turned to dietary supplements was to enhance muscle mass, with reported motivations ranging from 60.5% to 88.57%. Other key reasons included increasing strength and power (65.71%) and improving athletic performance and recovery (45.8% and 37.2%, respectively). These motivations align with global trends where supplements are widely consumed for body enhancement, strength-building, and overall health maintenance.^3,4^

A significant factor influencing supplement use was social influence. Many individuals relied on their peers (68.57% in one dataset, 54.3% in another) and gym instructors (27.14%) for recommendations. Advertisements, although present, played a comparatively minor role (ranging from 4.29% to 30.84%), suggesting that gym culture and social circles have a stronger impact on consumption behavior than traditional marketing. Previous research has also indicated that gym users often trust advice from friends and trainers more than medical professionals.^6,7^

One of the most concerning findings of this study was the significant knowledge gap surrounding dietary supplements. While 65.89% of respondents believed that supplements could contribute to cancer, only 15.42% were aware of their actual health risks. Furthermore, 39.7% of individuals lacked knowledge regarding supplement composition, proper dosages, and potential side effects, increasing the likelihood of severe health complications such as hepatotoxicity, nephrotoxicity, and cardiovascular issues.^8,9^

Adding to the problem, a considerable number of individuals relied on unverified sources such as social media (32.6%) for supplement-related information. This reliance on non-scientific sources can lead to misinformation, improper use, and unsafe consumption practices.^2^ There is an urgent need to bridge this knowledge gap through educational initiatives that promote accurate information and responsible supplement use.

To better understand how gym users perceive dietary supplements, this study applied the Health Belief Model (HBM), a widely used framework for assessing health-related behaviors:

Perceived Susceptibility: A significant portion of respondents (52.34%) believed that gym users were highly likely to gain muscle mass from supplements, with 41.12% strongly agreeing. This belief aligns with findings from global studies indicating that many individuals view supplements as essential for muscle growth.^3,4^. Also, the findings of our study indicate that a significant proportion of participants (66.36%) believe dietary supplements enhance muscle mass, contrasting with Saidi's (2018) interventional study, which found no significant improvement in lean body mass with protein-carbohydrate supplements. This discrepancy suggests a gap between perceived benefits and scientific evidence.^10^

Perceived Severity: Concerns about potential side effects were mixed, with 44.86% of respondents remaining neutral and 24.30% disagreeing that side effects were a concern. This suggests that a large number of gym-goers may underestimate the risks associated with supplement use, a trend also observed by Dickinson and MacKay (2014).^2^

Perceived Benefits: While 37.38% agreed and 18.22% strongly agreed that supplements help with muscle growth, 42.99% disagreed that they prevent exercise-related issues. This selective perception of benefits underscores the need for accurate information on both the advantages and limitations of dietary supplements.

Perceived Barriers: Cost was a major factor influencing supplement use, with 41.59% strongly agreeing that supplements are expensive. Additionally, 48.60% remained neutral when asked whether supplements suited their health needs, indicating uncertainty or a lack of proper guidance. This finding is consistent with global research showing that affordability and accessibility are significant barriers to supplement use, particularly in low- and middle-income countries.^11,12^

Cues to Action: External motivation played a significant role in supplement use. Parental support for supplement consumption was low, with 52.34% strongly disagreeing and 40.65% disagreeing that their parents encouraged their use. However, peer influence (31.78%) and advertisements (30.84%) had a relatively stronger impact, suggesting that gym culture and social environments, rather than family influence, drive supplement consumption behaviours.

The study also uncovered several misconceptions related to supplement use. For instance, 50.93% of respondents believed that any form of physical activity is beneficial after taking supplements, while 38.32% were unsure whether supplements alone could prevent low muscle mass. These misconceptions highlight the need for well-structured education programs that address false beliefs and promote evidence-based knowledge. Public awareness campaigns should focus on:

Correcting myths and misconceptions regarding supplement efficacy and safety.

Providing clear guidelines on safe and appropriate supplement use.

Encouraging consultation with healthcare professionals or certified nutritionists before starting any supplement regimen.

Given their strong influence, gym instructors should be actively involved in these educational initiatives to provide gym-goers with accurate and responsible guidance.

A critical concern raised in this study is the lack of proper regulatory oversight for dietary supplements in Nepal. A majority (58.88%) of respondents believed that there is no effective quality control system in place. This mirrors global concerns about supplement safety, particularly in countries with weak regulatory frameworks.^3,13^

A study conducted in South Asia found that nearly 30% of supplements available in unregulated markets contained undeclared substances, further emphasizing the need for stricter regulation.^14,15^ Ensuring proper oversight of the dietary supplement industry in Nepal could mitigate risks associated with unregulated products and protect consumer health.

## CONCLUSIONS

This study explored dietary supplement use among gym users in Nepal, finding that a significant portion relied on supplements, primarily for muscle enhancement. Social influences, such as peers and trainers, played a key role in consumption decisions. Many users held misconceptions about supplement risks and benefits, with concerns about potential long-term health effects.

Additionally, participants expressed skepticism about regulatory oversight of these products.
